# The Relation between the Dentist and the Surgeon1Read before the Alumni Association of the Dental Department of the University of Buffalo, February 20, 1902.

**Published:** 1902-06

**Authors:** Eugene A. Smith

**Affiliations:** Buffalo, N. Y., Adjunct professor of clinical surgery, University of Buffalo.


### The Relation Between the Dentist and the Surgeon.[1]

By EUGENE A. SMITH, M. D., Buffalo, N. Y.,
Adjunct professor of clinical surgery, University of Buffalo.

DENTISTRY is classed with surgery as a department of medicine, using the latter word in a broad sense. Surgeons are frank to say that dentistry is merely a department of surgery and they class dentists with specialists in other departments of surgery, such as genito-urinary and orthopedic surgeons. Dentists themselves incline more and more to assume the title of dental surgeons, and schools of dentistry under the name of colleges of dental surgery are graduating doctors of dental surgery.

As a branch of surgery it is natural that dentistry should keep pace with surgery in scientific progress. Discoveries in materials and methods originating from experiment and experience are appropriated by both, and in both are used to overthrow old methods of treatment, to improve results, and to offer relief and aid to the sufferer where formerly there was no help.

All general surgeons take pride in the advance which surgery has made in the last twenty-five years and we are hopeful of future progressive possibilities. Dentists also may justly pride themselves on the remarkable progress of dentistry, especially

---

1. Read before the Alumni Association of the Dental Department of the University of Buffalo, February 20, 1902.

in our own country in the past quarter of a century. Dental colleges, especially in our State of New York, have raised their standards of admission, have developed new departments, and have adopted graded courses; in short, they are graduating dental surgeons who are scientifically equipped to deal with the injuries and diseases of the mouth cavity, the jaws, and the teeth. The world has traveled far on the path of progress since the barber was the surgeon and a man with a pinchers was a dentist. Scott's description of a dentist does not excel in horror the word pictures of a surgeon in works less than one hundred years old. But we may expect a far nobler word painting when in the future some gifted author arises to do justice to the modern ideal practitioner in each profession.

Theoretically, surgery and dentistry, while closely allied as branches of medicine, cover distinct fields of their own. Practically the relation of the surgeon and dentist is not so clearly defined that each may always safely work on his own ground, secure in feeling he does not trespass upon the field of the other. There is undisputed territory upon which each may maintain himself in firm independence. There is also disputed border-line ground between dentistry and surgery where each may work with reason; and finally, there is the class of cases of such debatable character that both surgeon and dentist should meet, consult, and work in mutual support and dependence for the welfare of the patient.

Theoretically surgeons should take a course in dentistry, and dentists should have a course in general surgery. Practically it would not make the surgeon a dentist, excepting in the mutilating branch of a dentist's work, nor would it make the dentist more than an oral surgeon. But it would enable each to see his chosen field of work in its relationship to the other and to grasp the perspective, so to speak, of sequelæ and possibilities outside of his own domain and well within the boundaries of his colleague. In my earlier years of general practice, I found it necessary to send many patients to the dentist for treatment, following out the plain indications of hygiene and prophylaxis. In the treatment of many diseases, notably the exanthemata, scurvy, tuberculosis, diabetes and syphilis, and in gastrointestinal disorders, such as stomatitis and gastritis, my therapeutics would have gone halting and lame without the aid of the dental surgeon. At times, under stress of circumstances, I took upon myself to discharge some of his duties for the sufferer whose teeth, gums or mouth needed attention, and I trust I did as well as the dentist does when he occasionally infringes upon the prerogatives of the physician, by prescribing for the general disease which determines the local condition of the mouth which he has under his treatment.

In the last few years as a general surgeon my attention has been more and more frequently called to cases in the treatment of which I have been associated with a dentist, or have sent the patient to a dentist, or the dentist has referred one to me. In tribute to the professional courtesy of my dental associates in these cases, I can testify to the uniform professional and gentlemanly treatment I received from them, and I trust they say as much of my conduct toward them.

The relationship between dentist and surgeon in the natural order of subjects is first to be discussed in considering anesthesia. Mankind owes to Morton, a dentist, the discovery of this priceless boon and in a large sense, both surgery and dentistry are its beneficiaries above all other branches of science. The earthy sting in what would otherwise be a heavenly thing is the ever present danger which time has shown is attendant upon the use of an anesthetic. As a surgeon I confess a sense of grave responsibility in my daily work with anesthetics. Granting a careful preliminary examination of the patient and an urgent need of operation requiring anesthesia, the knowledge of immediate and post-operative dangers of our anesthetics should weigh upon us all as a serious responsibility. When we regard anesthesia from this point of view, we must conclude that a skilled anesthetist, a surgeon, or the family physician of the patient, should officiate for the dental surgeon, when an operation requires ether or chloroform and in addition a preliminary physical examination should be made of the patient, except in a case of extreme urgency. It seems to me the professional anesthetist, with a good experience, should find the dentists able and anxious to keep his hours filled. Surgeons are in a different catagory, because their operating hours conflict in a way to keep several anesthetists busy at the same time, and many operations are of an emergent nature.

We now come to the discussion of sepsis, asepsis and antisepsis, upon the understanding of which modern surgery has made its notable advances. All will admit that dentistry has been quick to apply the principles of antisepsis in dental work, and with brilliant results comparable with those of surgery. It is open to question, however, whether dentistry realises the full value of asepsis. In dental journals and societies dentists are still devoting lengthy discussions to the relative advantages and disadvantages of antiseptic agents, as though antisepsis is the sum of all that is to be said after admitting the bacterial origin of sepsis.

It is true that the mouth itself can only be made comparatively sterile by antiseptics. It cannot be sterilised by boiling or by exposure to high grade of dry heat, but instruments and materials can be so treated, while hands can be covered with sterile rubber gloves, and a field of operation in a tooth can be cleaned and rendered aseptic. I am inclined to state that more dentists than surgeons can be found who disbelieve in asepsis and antisepsis in their work. Such men base their belief on the specious argument that they see no bad results from a neglect of aseptic and antiseptic precautions. Seldom can we find a surgeon now who attempts to argue against the bacterial theory of sepsis. At the most, some few surgeons are inclined to minimise the danger of sepsis.

As clinicians, surgeons and dentists have many unstable boundary lines in the broad field of sepsis. Alveolar abscess in hundreds of cases may begin and end as a dental problem purely, but in the occasional case in which an infection extends from such an abscess by the deep lymphatics to the deep cervical nodes, it becomes a problem that is distinctly surgical. More emphatically, the problem becomes surgical when the sepsis extends to the lingual glands, to the antrum of Highmore, or develops into Ludwig's angina, or a septicemia, or even a pyemia. Only recently a case of abscess of the sublingual gland occurred under my observation from extension of sepsis originating about the root of a filled tooth, and on several occasions, as in this case, a dental associate refused to consider a sinus from a chronic alveolar abscess as an indication of danger needing radical treatment. Caries, necrosis and osteomyelitis, due to sepsis or drugs, according to their limitations, may be within the field of dentist or surgeon, or may be excellent ground for both to meet upon in consultation to the benefit of the patient.

Dental journals devote much space to the discussion of the etiology of dental diseases, and much of this is fruitless, because the bacterial cause is not admitted, or imperfectly understood. Inflammation with destructive tendency, and hyperemia with reparative tendency, are still confusedly used interchangeably by dental pathologists with resulting obscurity of meaning. It must not be forgotten that dental pathology is merely the local application of the truths of general pathology, and unless dentists devote themselves specifically to experimental laboratory work in pathology and bacteriology, their views on the etiology of dental diseases should be made to harmonise with the known facts of bacteriology and pathology. Many dental discussions now are reminders of the days in medicine when the exciting cause of a disease was deduced from clinical data with resulting interesting but unreliable theories of its etiology.

Passing to the differential diagnosis of new growths, surgeons are practically agreed that the pathologist and not the clinician is the diagnostician of all but the most simple and uncomplicated tumors. Epulis in dentistry and surgery is a term covering a multitude of errors. Too often an innocent, granulation-like mass, is found under the microscope to be small round-celled sarcoma, a most malignant new growth. Recently a young lad came under my care in the hospital who had lost an upper canine tooth and who had in its site a fungoid mass of this kind. It had been taken first for an abscess, then for granulation. Benign tumors are not always difficult of operative removal, but tumors of the malignant type need radical operative treatment, especially when the disease is not recent in origin. It follows, therefore, that dental surgeons must be ready to resect the superior maxilla, or to resect part or all of the inferior maxilla, with such clearing out of glandular and nodular involvement as may be necessary, before surgeons can concede to them the right to attack sarcoma or carcinoma affecting the structures of the mouth.

When we turn now to orthodontia, prosthetic and operative dentistry, the dental surgeon is within the well-defined bounds of his profession, and the surgeon must yield all claim to special training in the treatment of cases coming under these headings. Congenital defects and malformations, vicious growth of teeth, results of traumatisms, loss of teeth, conditions requiring fillings, crown and bridge work, and prosthetic work in general, are conditions that, as a rule, are entirely within the bounds of conservative dentistry. In cleft palate general surgeons are accustomed to operate, but in this field much attention is given to the treatment devised and advocated by Truman W. Brophy, M. D., D.D.S. In the treatment of fracture of the jaw interdental splints are more often employed by surgeons than formerly. In my own practice I have frequently called in a dental colleague for advice and help in the treatment of these cases, and I am convinced that better results would be obtained if such practice were more generally followed. The septic feature in these cases, compound as these fractures usually are, is the main factor in surgical treatment. The prosthetic feature is purely dental, and, so far as interdental splint fixation is concerned, is in every way best managed by the dentist.

We may turn now to the recent action of the United States Government in appointing and assigning dental surgeons to duty in the army, under the direction of the Surgeon-General, as an evidence of the evolution of dentistry and of its relation to surgery. Dental surgeons are appointed to the rank and pay of assistant surgeons, and a graduate of the dental department of the University of Buffalo had the honor of passing the rigid examinations which secured for him one of the earliest appointments under the new law.

I served in command of a battalion of the 65th Regiment New York Volunteers at Camp Black and Camp Alger during the Spanish-American War, and I can testify to the need of dental surgeons in the army from personal experience as well as from observation of the dental troubles of the enlisted men. I myself had the misfortune to break loose a crown and bridge between two molars in May at Camp Black while chewing army food (not army beef, as I remember), and it was August before I had the crown and bridge replaced, while home on a furlough, by one of your faculty here in Buffalo. The sufferers among the men when they were driven to dentists were overcharged for inferior treatment by mediocre dentists who put up their "shacks," as we called the outlying shanties, near our camp lines. Under present regulations such injustice to our soldiers will no longer be permitted. Thus we see that dental surgeons are called upon to aid in insuring the marching power of the men, it being a military axiom that "an army marches on its belly," and we know good teeth insure a good digestion, in other words, is a good belly.

It must be admitted that some mutterings of discontent are beginning to be heard, regarding the partiality shown to the dental profession in the appointment of dental surgeons to medical rank and pay. Technical training without a medical foundation evidenced by the degree of doctor of medicine, it is argued, should not be placed on the level of a surgical training developed after a long course in general medicine. In the next few years this question will be more freely discussed and finally decided, and to me it seems to tend strongly toward a medical diploma for the dental surgeon before his special degree is given upon the completion of his course in dentistry. The dental surgeon will then fairly stand upon the plane of the specialist in gynecology, ophthalmology, orthopedy, laryngology and other special branches of surgery. When this day arrives, if not before, every hospital staff will include the name or names of dental surgeons, as I am convinced should now be the case. In Buffalo at present only one hospital staff contains an oral surgeon's name. On the Buffalo General Hospital staff appears the name of your dean, W. C. Barrett, M. D., D.D.S.

In conclusion it must not be forgotten that this paper is written on the relation between the dentist and the surgeon—*by a surgeon.* When this relationship between the surgeon and the dentist is some day discussed from the viewpoint of the dentist, by a dentist, some alteration of opinion here expressed may naturally be expected. But I trust that harmony between dentist and surgeon will still prevail, redounding to the peace of mind of both, and far more than this to the health and welfare of the patient whose mouth disorder makes him an object of interest and a patient for the practitioners in both professions.

1018 Main Street.

